# Effects of p53 and ATRX inhibition on telomeric recombination in aging fibroblasts

**DOI:** 10.3389/fonc.2024.1322438

**Published:** 2024-01-25

**Authors:** Ion Udroiu, Jessica Marinaccio, Antonella Sgura

**Affiliations:** Dipartimento di Scienze, Università “Roma Tre“, Rome, Italy

**Keywords:** cancer, checkpoint, helicase, oncogenesis, recombinase, senescence, telomeres

## Abstract

In order to avoid replicative senescence, tumor cells must acquire a telomere maintenance mechanism. Beside telomerase activation, a minority of tumors employs a recombinational mechanism called Alternative Lengthening of Telomeres (ALT). Several studies have investigated the potential ALT stimulation by inactivation of ATRX in tumor cells, obtaining contrasting results. Differently, since ALT can be viewed as a mechanism to overcome telomere shortening-mediated replicative senescence, we have investigated the effects of the inhibition of ATRX and p53 in aging primary fibroblasts. We observed that senescence leads to a phenotype that seems permissive for ALT activity, i.e. high levels of ALT-associated PML bodies (APB), telomeric damage and telomeric cohesion. On the other hand, RAD51 is highly repressed and thus telomeric recombination, upon which the ALT machinery relies, is almost absent. Silencing of ATRX greatly increases telomeric recombination in young cells, but is not able to overcome senescence-induced repression of homologous recombination. Conversely, inhibition of both p53 and ATRX leads to a phenotype reminiscent of some aspects of ALT activity, with a further increase of APB, a decrease of telomere shortening (and increased proliferation) and, above all, an increase of telomeric recombination.

## Introduction

In order to achieve immortality, cells must acquire a telomere maintenance mechanism, otherwise they will undergo telomere shortening at each cell division, and finally reach telomere shortening-induced senescence (1). This is often achieved by reactivation of telomerase, through overexpression of oncogenes like MYC or mutations of the TERT (the gene encoding the catalytic subunit of telomerase) promoter (2, 3). Nevertheless, several tumors and immortal cell lines show an Alternative Lengthening of Telomeres (ALT) recombinogenic mechanism (4).

Among the studies on ALT establishment, much attention has been paid to the role of ATRX as an inhibitor of this mechanism. In fact, loss of this gene has been showed to be highly associated with the ALT phenotype in pancreatic neuroendocrine tumors (5), astrocytomas (6), glioblastomas (7) and sarcomas (8). The main hypothesis is that without ATRX-mediated deposition of H3.3 histone telomeres become euchromatic and prone to recombination (9, 10), although the actual euchromatic/heterochromatic status of telomeres has been disputed (11–13). Nonetheless, loss of ATRX has been proposed to trigger ALT through increase (14) or decrease (15) of telomeric cohesion, replication fork collapse at telomeric G4 structures (16), and defects in replication fork restart (17). Apart from its role as an ALT suppressor, ATRX has been showed to be critical for the formation of senescence-associated heterochromatic foci (SAHF) (18). These are areas of transcriptionally silent and compacted chromatin that maintain the cell in a non-proliferating state, as the E2F transcription factors cannot bind to the compressed and therefore inaccessible chromatin, which prevents transcription of S-phase cell cycle related genes (19).

On the other hand, less attention has been paid to the role of p53 in the establishment of ALT. This despite the fact that while not all ALT cell lines are ATRX-deficient, almost all of them are p53-deficient (20). Nonetheless, it is known that p53 inactivation leads to an increase of HR (21). In particular, Arias-Lopez et al. (22) showed that p53 downregulates RAD51 expression and that p53 inhibits RAD51 foci formation in response to double-strand breaks (DSB). It was also shown that phosphoserine-15 form of p53 colocalizes with RAD51 (23) and represses homologous recombination (HR) (24). This activity of p53 is independent from its cell cycle regulatory capacity (24–26). Moreover, Akter et al. (27) showed that expression of RTEL1, FANCD2, BLM, WRN, and RECQL4 (which are all protein essential for ALT activity) is upregulated by the inactivation of p53 and Jaber et al. (28) demonstrated that p53 downregulates the Fanconi anaemia DNA repair pathway (including BLM, FANCD2, FEN1, GAR1 and RECQL4).

Having worked extensively on fibroblasts, we noted a decline on DSB repair as cells age (29). Sabin et al. (30) demonstrated that this reduced efficiency is present in both proliferative and non-proliferative cells, thus not merely linked to cell cycle delay/exit. Indeed, Collin et al. (31) evidenced that during cell aging there is a RB-mediated transcriptional repression of DNA repair genes. In particular, it was observed a threefold reduction in non-homologous end-joining (NHEJ) efficiency (32) and a 38-fold reduction in the efficiency of HR repair (33) between young and pre-senescent cells.

Since ALT can be viewed as a mechanism to overcome telomere shortening-mediated replicative senescence, we wanted to investigate the effects of the inhibition of p53 and ATRX (presumed to be the main repressors of ALT) not only in young, but also in aging primary fibroblasts (including both old, slowly proliferating cells and older, growth arrested ones, i.e. senescent cells). We have not used p53-deficient fibroblast lines (e.g., from Li–Fraumeni syndrome patients), because these are genomically unstable and accumulate secondary mutations. As an alternative, we used Pifithrin-α, a chemical that specifically inhibits phosphorylation of p53 at serine 15 (34), thus preventing repression of HR (24). In this study, we used pifithrin and/or small interfering RNA against ATRX, in order to investigate how p53 and/or ATRX inhibition affect age-related changes in telomeric recombination.

## Material and methods

### Chemicals and cell cultures

Pifithrin-α (pft) was purchased from Sigma-Aldrich (Germany). Stock solutions of pft (34 mM) were prepared in DMSO and stored in small aliquots at −20°C. Growth medium was supplemented with pft from the stock solutions as needed.

Human Fetal Foreskin Fibroblasts (HFFF2) (ECACC, UK) were grown in D-MEM High Glucose (4.5 g/l) (Euroclone, Italy), supplemented with 10% fetal bovine serum, 100 units/ml penicillin, 100 ug/ml streptomycin, and 2 mM L-glutamine. Cells were maintained in a humidified incubator at 37°C, with 95% relative humidity and 5% CO2.

Cells from a single batch at 25 population doublings (PD) were used in all experiments. Cells were grown and sub-cultured for 12 days, when a first round of experiments (each in triplicate) was performed (termed “young”, “Y”). Sub-cultures were furtherly grown for two months, when some experiments were performed (termed “old”, “O”). From this time on, 3 sub-cultures were grown untreated, 3 were chronically treated with pft, 3 were chronically treated with siRNA against ATRX, 3 were chronically treated with a scramble siRNA and 3 were chronically treated with pft and siATRX. After 20 days, a final round of experiments (termed “senescent”, “S”) was performed on all sub-cultures.

From a biological point of view, the effects of chronic treatment are the most interesting; however, the effects on young cells could only be observed by short-term treatment (i.e., avoiding aging during a chronic treatment). Therefore, in order to allow another direct comparison, short-term treatment (see below) was performed also on the untreated senescent sub-cultures. Summing up, the timepoints consisted of: young, short-term treated; old, short-term treated; senescent, chronically treated and senescent, short-term treated.

### Small interfering RNA and transfections

Pre-designed small interfering RNA (siRNA) against human ATRX mRNA (siATRX#2 5’-UAUAGAAUUCUGAUCAUCA-3’) and a scrambled siRNA (5’-GAUUGAAGACUGUAUCAUU-3’) were purchased from Sigma-Aldrich. Cells were transfected with Lipofectamine RNAiMAX (Thermofisher, Italy) according to the manufacturer’s instructions twice: the first transfection during the seeding and the second one 24 hours later. In the case of chronic treatments, a third transfection was performed after another 72 hours and after 72 hours more the protocol began again (for a total of 3 transfections per week).

### Treatment protocols

**Figure 1:**
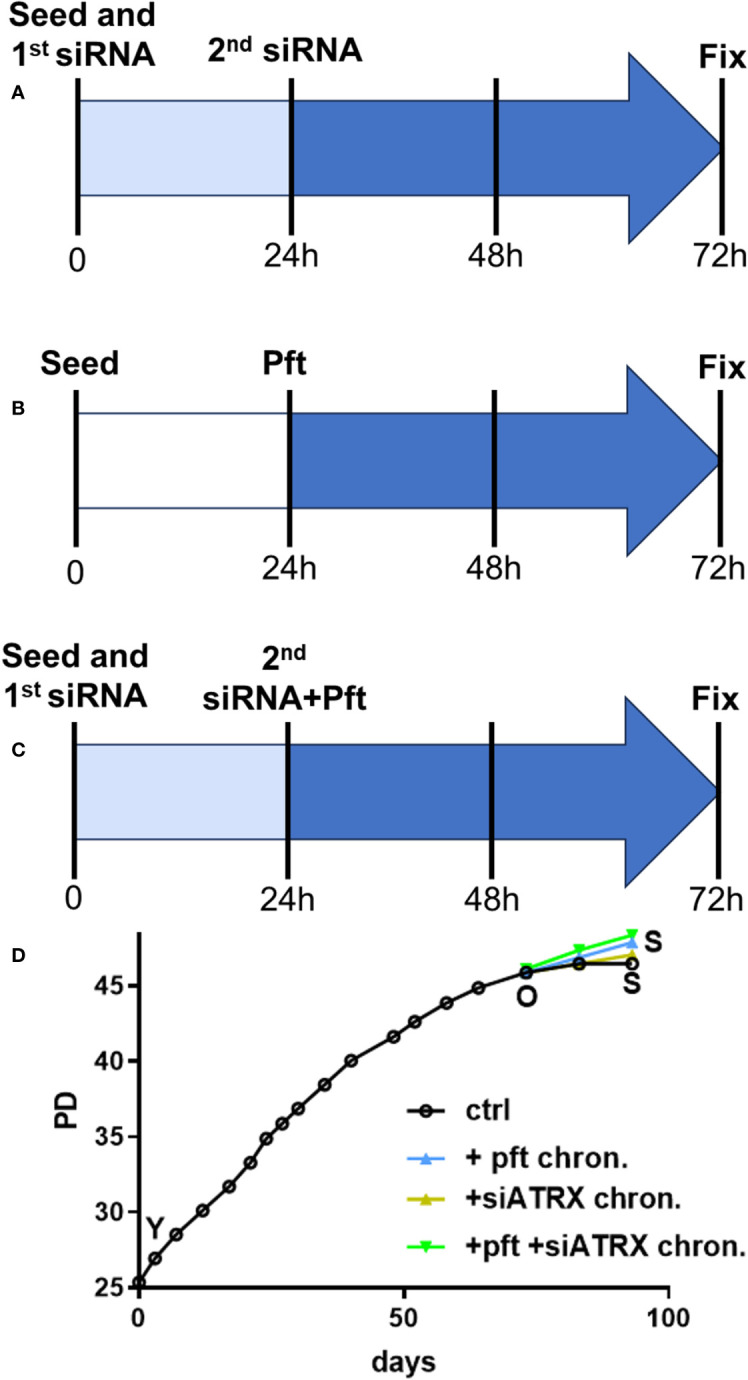
Treatment protocols. **(A)** In order to reach full effect of siATRX treatment, transfections were performed twice (during seeding and 24 hours later); therefore, treatment time was from 24 to 72 hours post-seeding (48 hours in total). **(B)** Cells were treated with 10 µM pft from 24 to 72 hours post-seeding (48 hours in total). **(C)** same as A, but pft was added immediately after the second transfection. **(D)** Growth curves of sub-cultures with different treatments; PD: population doublings; Y: young; O: old; S: senescent; ctrl: controls; + pft chron.: chronically treated with pft; + siATRX chron.: chronically treated with siATRX; + pft + siATRX chron.: chronically treated with pft and siATRX.

Cells were seeded on different petri dishes, depending on the assay to be performed (see below) at a concentration of 7000 cells/cm2. In the case of siATRX treatment, transfections were performed during seeding and 24 hours later (see above). In the case of pft treatment, the compound at a concentration of 10 µM (34; 35) was added 24 hours after seeding. In the case of combined treatment, pft was added immediately after the second transfection. Cells were fixed or harvested 72 hours after seeding, giving 48 hours of treatment in every case (**Figure 1A-C**).

For pft chronic treatment, the fresh medium containing pft was added twice a week.

Evalauation of p53 inhibition and ATRX silencing is reported in **Supplemenary Material** (**Figures S1, S2**).

### Indirect immunofluorescence

Cells were fixed with ice-cold methanol for 20 minutes, then blocked in PBS/BSA 1% for 30 minutes at room temperature. Slides were then incubated for one hour at 37°C with different primary antibodies: for ATRX foci, mouse anti-ATRX (Santa Cruz Biotechnology, USA) and human anti-kinetochore (Antibodies Inc., USA) antibodies; for ALT-associated PML bodies (APB), mouse anti-PML (Santa Cruz Biotechnology, USA) and rabbit anti-TRF1 (Santa Cruz Biotechnology, USA) antibodies; for RAD51 and γH2AX colocalization, rabbit anti-RAD51 (Santa Cruz Biotechnology, USA) and mouse anti-γH2AX (Millipore, USA) antibodies; for TRF1 and γH2AX colocalization, rabbit anti-TRF1 (Santa Cruz Biotechnology, USA) and mouse anti-γH2AX antibodies; for RAD51 and TRF1 colocalization, rabbit anti-RAD51 (Santa Cruz Biotechnology, USA) and mouse anti-TRF1 (GeneTex, USA) antibodies; for nucleoplasmic bridges (NPB), rabbit anti-TRF1 (Santa Cruz Biotechnology, USA) antibody. Successively, slides were incubated for 1 hour at 37°C with either anti-mouse Alexa 546 (Invitrogen, USA) and anti-rabbit Alexa 488 (Invitrogen) or anti-mouse Alexa 488 (Invitrogen), anti-rabbit Alexa 546 (Invitrogen) and Cy3-conjugated anti-human (Jackson ImmunoResearch, USA) antibodies. Coverslips were mounted with DAPI (Sigma-Aldrich, USA) diluted in antifade solution (Vectashield, Vector Laboratories, USA) to a final concentration of 1 μg/mL. Only in the case of samples used for senescence-associated heterochromatic foci (SAHF), DAPI concentration was 0.2 μg/mL (36). Cells were analyzed with an Axio Imager M1 fluorescent microscopy (Carl Zeiss, Germany). For samples stained with antibodies for γH2AX, RAD51 and TRF1, each image was acquired on 10 different focal planes (spaced by 0.2 μm) on the z-axis; colocalization was scored only if signals of both fluorophores were in focus on the same plane. The frequencies of foci per cell were scored in 100 nuclei in at least three independent experiments.

Cells were classified as SAHF-positive or negative depending on whether DAPI staining in the nucleus showed foci or was homogeneous (36) (although this may seem subjective, no intermediate staining was observed).

### Collection and fixation of chromosome spreads

Seventy-two hours after seeding, chromosome spreads were obtained following 30 minutes incubation in 30 μM Calyculin-A (Wako Chemicals, Japan). Spreads of prematurely condensed chromosomes were prepared by a standard procedure consisting of treatment with a hypotonic solution (75 mM KCl) for 28 minutes at 37°C, followed by fixation in freshly prepared Carnoy solution (3:1 v/v methanol/acetic acid). Cells were then seeded onto slides and utilized for cytogenetic analysis.

### Chromosome orientation-FISH analysis

Twenty-four hours before fixation, cells were treated with 5-bromo-2’-deoxyuridine (BrdU, Sigma-Aldrich) at a final concentration of 25 µM. Chromosome spreads were obtained as described in the previous section. After 24 hours at room temperature, slides with chromosome spreads were rinsed with PBS and then treated with 10 mg/mL DNAse-free RNAse A (Sigma-Aldrich) at 37°C for 15 minutes. After 5 minutes rinsing in PBS, slides were stained in 0.5 mg/mL Hoechst 33258 (Sigma-Aldrich) at room temperature for 30 minutes. Slides were then washed with saline-sodium citrate (SSC) pH 7.0 and exposed to 365 nm UV light for 4 h. Then, slides were placed in a 200 U/μL exonuclease III (Promega, Italy) solution and incubated at 37 °C for 30  minutes. After rinsing in PBS for 5 minutes, slides were washed in SSC at 45 °C for 20  minutes and dehydrated through graded alcohols. Slides were then hybridized using two different probes: a (TTAGGG)3 probe labelled with FITC and then a (CCCTAA)3 probe labelled with Cy3 (Panagene, Korea). After hybridization, slides were washed twice for 15 min in 70% formamide, 10 mm Tris pH 7.2, and 0.1% PBS/BSA, followed by three 5-min washes in 0.1 m Tris pH 7.5, 0.15 m NaCl, and 0.08% Tween 20. Slides were then dehydrated with an ethanol series and air dried. Finally, slides were counterstained with DAPI in antifade solution. Images were captured with an Axio Imager M1 fluorescent microscopy (Carl Zeiss, Germany), using filters for DAPI (420 nm emission), Cy3 (590 nm emission), and FITC (500 nm emission), equipped with a CCD camera and analyzed by ISIS software (MetaSystems).

T-SCE (Telomere Sister Chromatid Exchanges) events were scored when a double signal was visible with both the Cy3 and FITC probes. Experiments were repeated three times and 10 chromosome spreads were analyzed for each line and condition.

### Telomeric quantitative FISH

Slides with chromosome spreads (obtained as described above) were left at room temperature for 48 hours, and then were rinsed with PBS pH 7.4 and fixed in 4% formaldehyde for 2 minutes. After two rinses in PBS, the slides were dehydrated through graded alcohols. Subsequently, slides and probes (Cy3-linked telomeric and chromosome 2 centromeric Peptide Nucleic Acid probes, Panagene) were co-denatured at 80°C for 3 minutes and hybridized for 2 hours at room temperature in a humidified chamber. After hybridization, slides were washed with the same solution used in CO-FISH technique (see previous section). Slides were counterstained with DAPI in antifade solution. Chromosome spreads were captured with an Axio Imager M1 fluorescent microscopy (Carl Zeiss) equipped with a CCD camera, using filters for DAPI (420 nm emission) and Cy3 (590 nm emission). Using ISIS software, telomere lengths were calculated as the ratio between the total telomeres fluorescence (T) and the fluorescence of the centromeres of the two chromosomes 2 (C). Data were expressed as a percentage (T/C%). At least 10 metaphases were analyzed for each sample and experiments were repeated at least three times.

### Statistical analyses

For each endpoint analysed, the statistical unit was the experiment (and not the single cell or chromosome spread). All data were analysed using the two samples *t* test. The level of significance was established at p<0.05. No experiment was discarded.

## Results

### Cell growth and aging

We first investigated the effects of the different treatments on the aging phenotype. Cell cultures showed a gradual decrease of growth potential (**Figure 1D**) until they reached 45.9 population doublings (PD). This timepoint was termed “old” (O) and thereafter control (i.e, scramble-treated), pft-treated, siATRX-treated and pft+siATRX-treated subcultures were carried on until the end of the experiment (termed S for senescent). In the following 10 days, control cells showed minimal growth, reaching 46.5 PD and without further growth in the subsequent 10 days. Cells treated with siATRX reached 47 PD at the end of the last 20 days, while pft-treated and pft+siATRX-treated reached 47.9 PD at the end of the experiment.

Beside cell growth, we wanted to investigate if the different treatments had any effect on some age-related markers. Having worked extensively with fibroblasts for many years, we are used to observe polyploid and binucleated cells in senescent cultures: although not widely known, this phenomenon has been observed for many decades (reviewed in (37)). While binucleated and 4N cells were absent among young fibroblasts, both appeared in old cells and further increased in senescent cells (**Figure S3** in **Supplementary Material**). No treatment had any effect on the frequencies of binucleated and 4N cells.

Examining ATRX foci, we first observed an increase with aging. However, we noted higher numbers of ATRX foci only in cells with very large nuclei. Subsequent discrimination between 2N and 4N cells (using anti-kinetochore antibodies) revealed no increase of ATRX foci in 2N cells (**Figure S3** in **Supplementary Material**) and a circa two-fold higher level in 4N cells (which were present only in old and senescent cells). Treatment with siATRX and pft+siATRX completely removed ATRX foci (confirming the efficiency of siRNA). Short-term (last 72 hours) and chronic (last 20 days) pft treatment reduced ATRX foci, but not in a significant manner.

**Figure 2:**
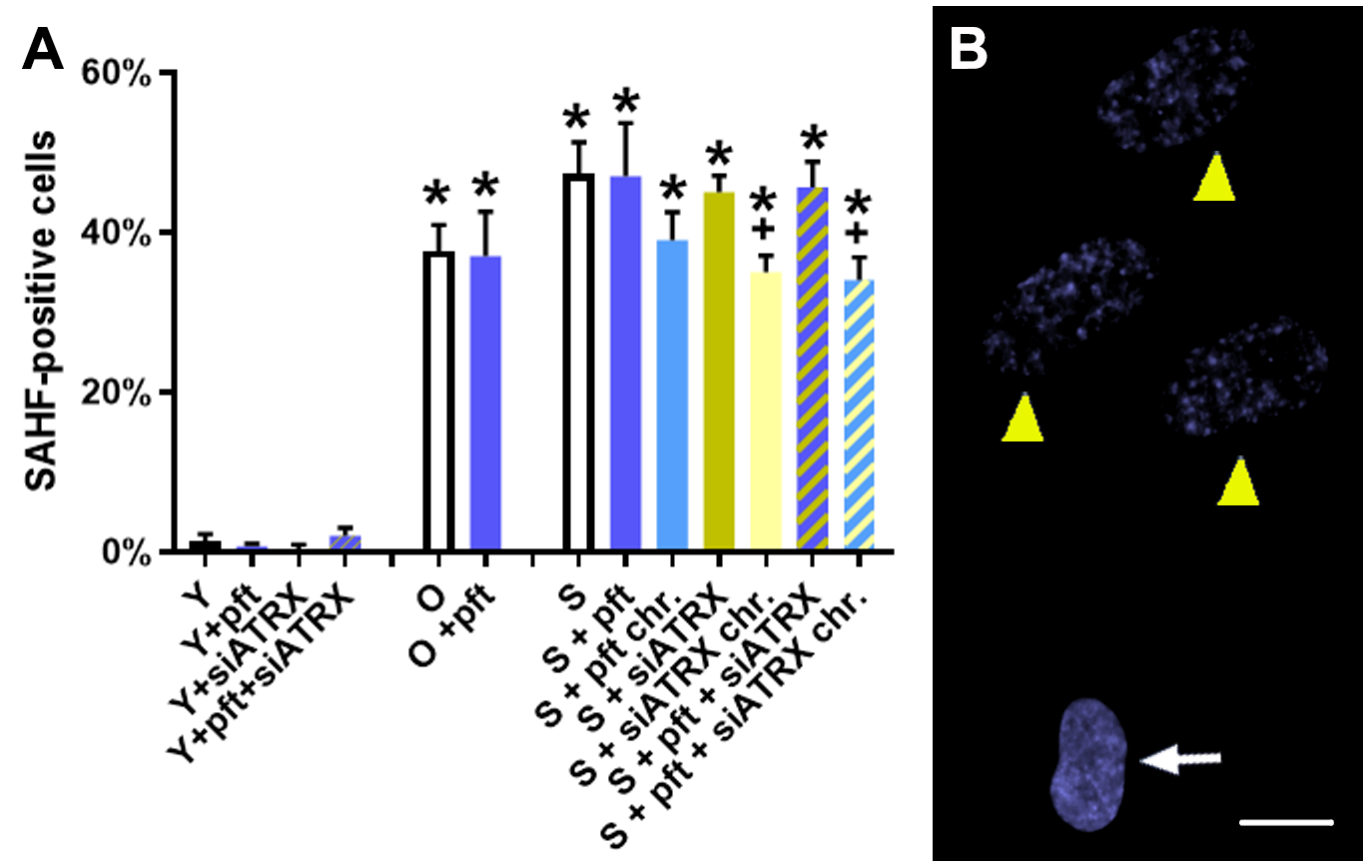
Markers of aging in fibroblasts. **(A)** Percentage of cells positive for senescence-associated heterochromatic foci (SAHF). *: significant compared to young cells; +: significant compared to matched-age control cells. **(B)** Representative image of low concentration DAPI-stained senescent control cells, showing a normal nucleus (arrow) and nuclei with SAHF (yellow arrowhead). Scale bar, 10 µm.

In old and senescent cells, we observed, as expected, a significant (p=0.0004 and p=0.0003, respectively) increase of senescence-associated heterochromatic foci (SAHF), compared to young cells (**Figure 2**). Interestingly, chronic treatment for the last 20 days with siATRX and pft+siATRX caused a significant decrease (p=0.0422, both) of SAHF compared to control senescent cells.

### Combined p53 and ATRX inhibition increases ALT-associated PML bodies levels

**Figure 3:**
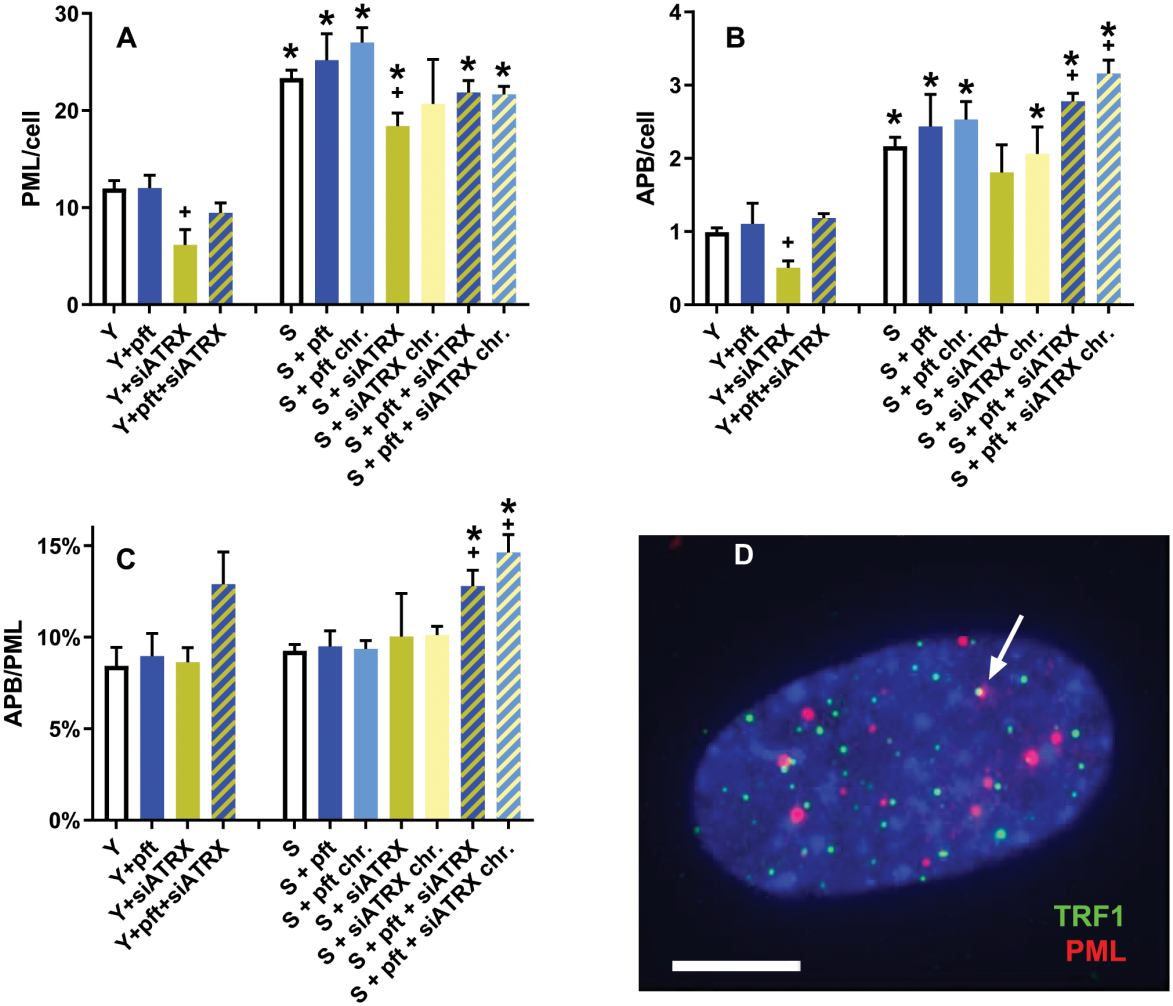
APB in aging fibroblasts. **(A)** Frequencies of PML foci. **(B)** Frequencies of APB. **(C)** ratio between APB and PML foci. **(D)** Representative image of a young control cell, showing telomeres (green), PML foci (red) and two colocalizing signals (arrow) indicating an APB; DAPI in blue. *: significant compared to young cells; +: significant compared to matched-age control cells. Scale bar, 5 µm. Single-channel photos are presented in **Supplementary Material** (**Figure S4**).

Since ALT cell lines display APB (i.e., telomeric PML), we wanted to investigate if cellular aging, p53 inhibition and ATRX inhibition influence the levels of APB (**Figures 3A-D**).

In young cells, siATRX treatment significantly (p=0.03) decreased PML foci, whereas pft and pft+siATRX treatment had no significant effect (**Figure 3A**). In senescent cells, there was a significant increase compared to young ones (p=0.0006). Short-term and chronic treatment of senescent cells increased PML foci compared to control senescent cells, but not in a significant manner. Treatments with siATRX and pft+siATRX (both short-term and chronic) decreased PML foci compared to control senescent cells, but the difference was significant only in siATRX-treated cells (p=0.0338).

Similarly, we observed that in young cells, telomeric PML foci (APB) were significantly decreased (p=0.0112) only in siATRX-treated cells (**Figure 3B**). In senescent cells, there was a significant increase compared to young ones (p=0.0009). Among the various treatments performed on senescent cells, only pft+siATRX (both short-term and chronic) exerted a significant effect (p=0.0203 and p=0.0108, respectively) compared to control senescent cells, increasing the frequencies of APB.

The similarity of the results between PML and APB was reflected by the fact that the ratio between APB and PML was nearly constant in almost all the samples (**Figure 3C**). However, in young cells treated with pft+siATRX there was a non-significant increase and in pft+siATRX-treated senescent cells, both short-term and chronic, the increase was significant (p=0.0184 and p=0.0064, respectively).

### P53 inhibition counteracts age-related decline of RAD51

In order to investigate the effects of aging and of the various treatments on homologous recombination (HR), we evaluated the levels of genomic and telomeric γH2AX (marker of DNA double-strand breaks) and RAD51 (marker of HR) foci.

**Figure 4:**
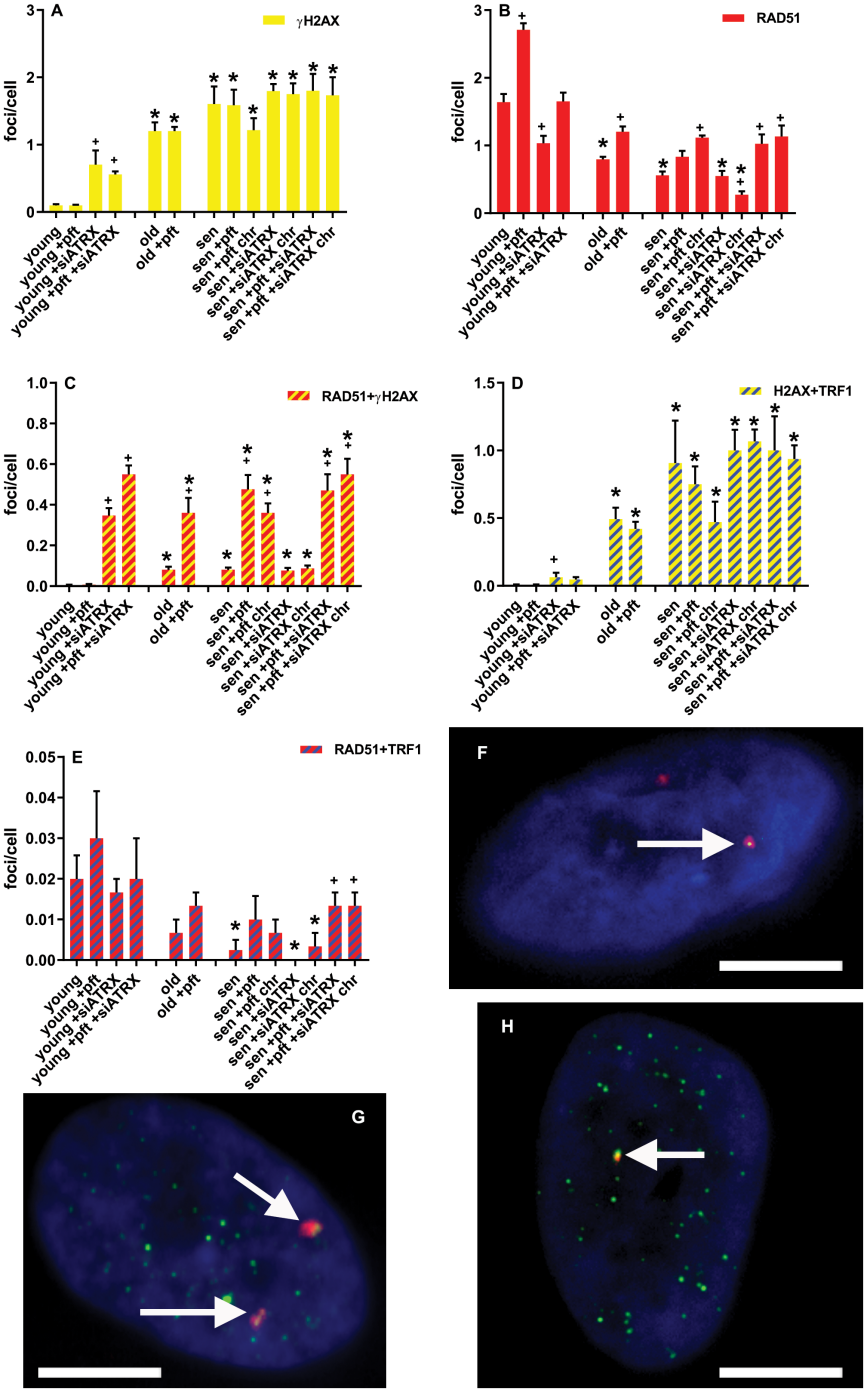
DNA damage and repair foci. **(A)** Mean frequencies of γH2AX foci. **(B)** Mean frequencies of RAD51 foci. **(C)** Mean frequencies of RAD51+γH2AX colocalizing foci. **(D)** Mean frequencies of γH2AX+TRF1 colocalizing foci. **(E)** Mean frequencies of RAD51+TRF1 colocalizing foci. sen: senescent; chr: chronic treatment. *: significant compared to young cells; +: significant compared to matched-age control cells. F-H: Representative images of pft-treated senescent cells stained with antibodies for γH2AX (red) and RAD51 (green) **(F)**, γH2AX (red) and TRF1 (green) **(G)**, RAD51 (red) and TRF1 (green) **(H)**. Arrows show colocalizations. Scale bar, 5 µm. Single-channel photos are presented in **Supplementary Material** (**Figure S5**).

In young cells, pft treatment did not induce DNA damage (**Figure 4A, D**), but significantly increased (p=0.0023) RAD51 foci (**Figure 4B**). ATRX silencing, on the other hand, significantly increased (p=0.0452) γH2AX foci (**Figure 4A**) and TRF1+γH2AX colocalizing foci (p=0.0123, **Figure 4D**). This was accompanied by a significant increase (p=0.0008) of RAD51+γH2AX colocalizing foci (**Figure 4C**). Nonetheless, the total level of RAD51 foci (**Figure 4B**) was significantly decreased (p=0.0205). In cells treated with pft and siATRX, we observed a significant increase (p=0.0004) of γH2AX foci, although smaller than the one induced by siATRX alone (**Figure 4A**), and an increase, but not significant (p=0.0898), of TRF1+γH2AX colocalizing foci (**Figure 4D**). Despite the fact that the total level of RAD51 foci was not different from the one of control cells (**Figure 4B**), there was a significant increase (p=0.0002) of RAD51+γH2AX colocalizing foci (**Figure 4C**), and all γH2AX foci were colocalizing with RAD51.

In old cells, there was a significant increase (p=0.0012) of γH2AX foci (**Figure 4A**) and TRF1+γH2AX colocalizing foci (p=0.0042, **Figure 4D**), compared to the young ones. Although there was a significant increase (p=0.008) of RAD51+γH2AX colocalizing foci (**Figure 4C**), the total level of RAD51 foci was significantly (p=0.0027) decreased (**Figure 4B**). In old cells treated with pft, there was a significant increase of the total level of RAD51 foci (**Figure 4B**) and RAD51+γH2AX colocalizing foci (**Figure 4C**), compared to old control cells (p=0.0094 and p=0.0205, respectively).

In senescent cells, there was a significant increase (p=0.0045) of γH2AX foci (**Figure 4A**) and TRF1+γH2AX colocalizing foci (p=0.0458, **Figure 4D**), compared to the young ones. Although there was a significant increase (p=0.0031) of RAD51+γH2AX colocalizing foci (**Figure 4C**), the total level of RAD51 foci was significantly (p=0.0013) decreased (**Figure 4B**). Also the level of RAD51+TRF1 colocalizing foci significantly (p=0.0272) decreased (**Figure 4E**). Pft treatment significantly increased (p=0.0049) the level of RAD51+γH2AX colocalizing foci (**Figure 4C**). Similarly, in cells chronically treated with pft there was a significant increase (p=0.0045) of RAD51+γH2AX colocalizing foci (**Figure 4C**), but also a significant increase (p=0.001) of the total level of RAD51 foci (**Figure 4B**). Treatment with siATRX did not induce any significant change, while in cells chronically treated with siATRX the total level of RAD51 foci was significantly (p=0.0185) decreased (**Figure 4B**). In cells treated with pft and siATRX there was a significant increase (p=0.0348) of the total level of RAD51 foci (**Figure 4B**), a significant increase (p=0.0086) of RAD51+γH2AX colocalizing foci (**Figure 4C**), and a significant increase (p=0.04) of RAD51+TRF1 colocalizing foci (**Figure 4E**). Similarly, in cells chronically treated with pft and siATRX there was a significant increase (p=0.0285) of the total level of RAD51 foci (**Figure 4B**), a significant increase (p=0.0037) of RAD51+γH2AX colocalizing foci (**Figure 4C**), and a significant increase (p=0.04) of RAD51+TRF1 colocalizing foci (**Figure 4E**).

### P53 and ATRX inhibition differently affect telomeric recombination

**Figure 5:**
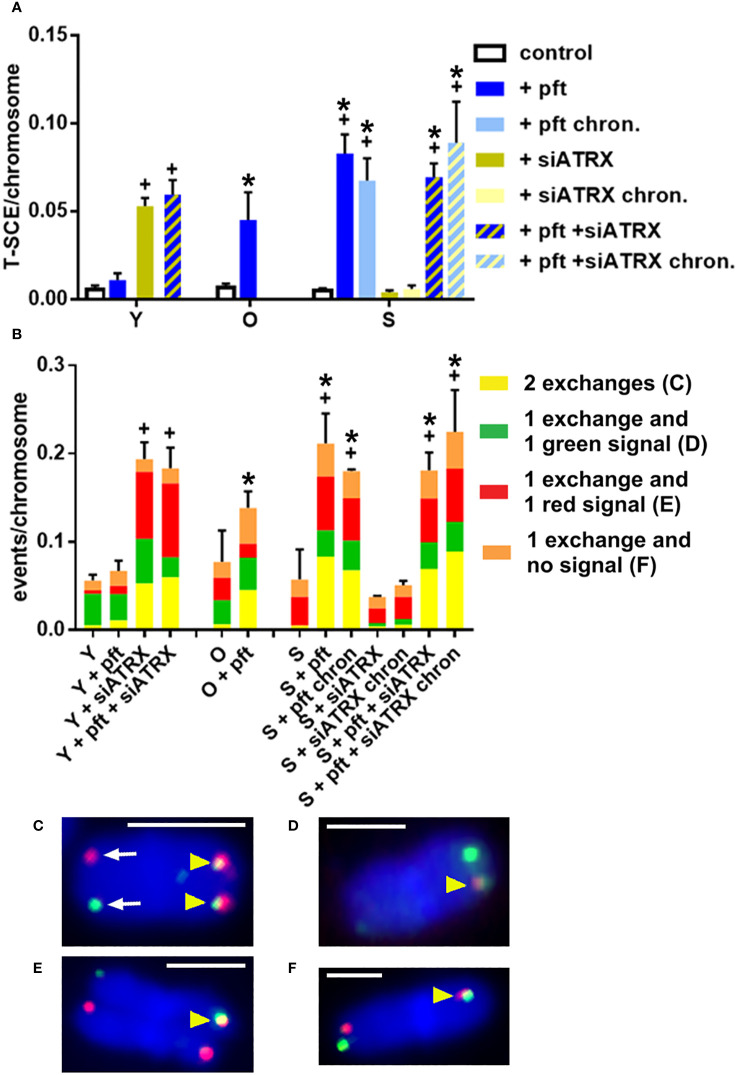
Telomeric recombination after different treatments. Telomeric sister chromatid exchanges (T-SCE) were scored using the two-color CO-FISH technique, with strand-specific C-rich (red) and G-rich (green) telomeric probes. **(A)** Mean frequencies of equal T-SCE in different samples. **(B)** Mean frequencies of different telomeric recombination types. **(C)** Equal T-SCE, consisting of double signals (yellow arrowheads) on each chromatid (exchange + exchange); arrows show normal telomeres. **(D)** Double signal (arrowhead) on a chromatid and single green signal on the other one (exchange + green). **(E)** Double signal (arrowhead) on a chromatid and single red signal on the other one (exchange + red). **(F)** Double signal (arrowhead) on a chromatid and no signal on the other one (exchange + no signal). *: significant compared to young cells; +: significant compared to matched-age control cells. Scale bar, 2 µm. Single-channel photos are presented in **Supplementary Material** (**Figure S6**).

After measuring HR activity at telomeres as presence of telomeric RAD51, we next analyzed T-SCE, which are a product of telomeric recombination and are largely accepted as an ALT hallmark (Henson & Reddel (20)). In two-color Chromosome-Oriented (CO)-FISH, T-SCE are usually scored as pairs of sister telomeres that both show a double hybridization signal (i.e., both leading and lagging strands, **Figure 5C**), called ‘equal T-SCE’. However, we also scored other types of combinations: a double signal on a telomere and a lagging strand signal (**Figure 5D**) or a leading strand signal (**Figure 5E**) or no signal (**Figure 5F**) on the other one. We scored all these types together as ‘total T-SCE’.

In young cells, treatment with siATRX and pft+siATRX induced a significant increase (p=0.0019 and p=0.0048, respectively) of equal T-SCE frequencies (**Figure 5A**) and a significant increase (p=0.0047 and p=0.0018, respectively) of total T-SCE frequencies (**Figure 5B**). In old cells treated with pft there were not significant increases of T-SCE frequencies. In senescent cells treated with pft there was a significant increase of equal and total T-SCE frequencies (p=0.002 and p=0.0329, respectively), compared to control cells (**Figure 5**). Similarly, chronic treatment with pft induced a significant increase of equal and total T-SCE frequencies (p=0.0079 and p=0.0231, respectively). Treatment with siATRX, both short-term and chronic, did not induce significant changes. Short-term and chronic treatment with pft+siATRX induced a significant increase (p=0.0013 and p=0.0227, respectively) of equal T-SCE frequencies and a significant increase (p=0.0358 and p=0.0453, respectively) of total T-SCE frequencies (**Figure 5**).

### Cellular aging increases sister telomere fusion

Since telomeric cohesion has been proposed by some authors to be linked with telomeric recombination and ATRX deficiency, we wanted to investigate the effects of aging and of the various treatments we used.

**Figure 6:**
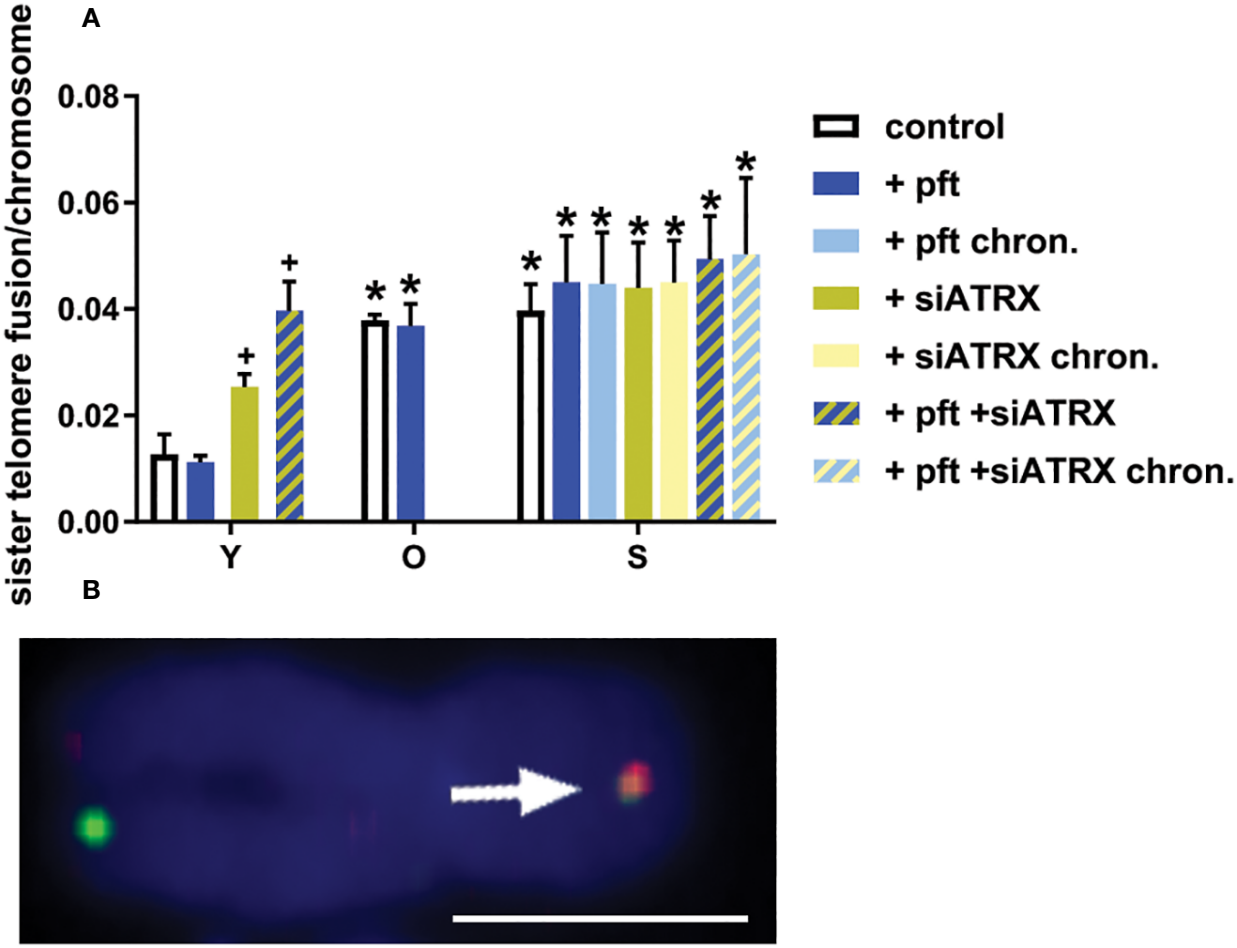
Sister telomere fusions after different treatments. **(A)** Mean frequencies of sister telomere fusions (STF) in different samples. *: significant compared to young cells; +: significant compared to matched-age control cells. **(B)** Representative image of a chromosome (from a senescent control sample) stained with two-color CO-FISH, showing a STF (arrow). Scale bar, 2 µm. Single-channel photos are presented in **Supplementary Material** (**Figure S7**).

In young cells, treatment with siATRX and pft+siATRX induced a significant increase (p=0.049 and p=0.0083, respectively) of sister telomere fusion (STF, **Figure 6**). In old and senescent cells there was a significant increase of STF (p=0.0026 and p=0.0067, respectively) compared to the young ones. No treatment had any significant effect in these cells.

To further confirm these observations, we analysed nucleoplasmic bridges (NPB) in binucleated cells and dicentrics in chromosome spreads, obtaining results in agreement with STF frequencies (**Figure S8** in **Supplementary Material**).

### Effects on telomere shortening

Since telomeric recombination is considered to be the mechanism by which ALT cells elongate their telomeres (Henson and Reddel (20)), we investigated if the increases in telomeric recombination we observed affected telomere length.

**Figure 7:**
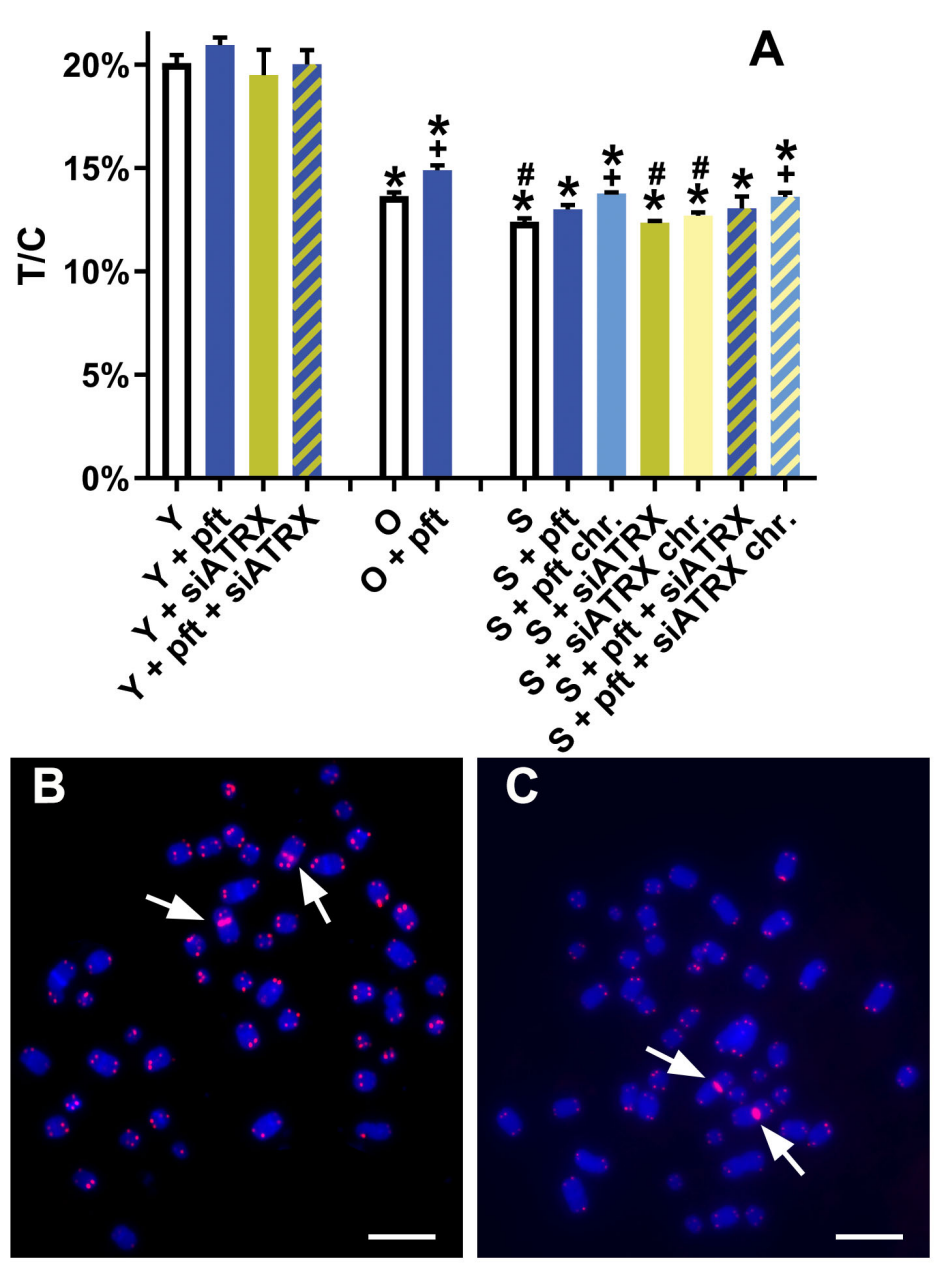
Telomere lengths after different treatments. **(A)** T/C: ratio between the total telomeres fluorescence (T) and the fluorescence of the centromere of chromosome 2 (C). *: significant compared to young cells; #: significant compared to old cells; +: significant compared to matched-age control cells. **(B, C)** Representative chromosome spreads of young **(B)** and senescent **(C)** control cells, showing centromeres of chromosome 2 (arrows) and telomeres stained in red. Scale bar, 10 µm.

No treatment significantly changed telomere length among young cells (**Figure 7**). Among old fibroblasts, control cells showed telomeres significantly shorter than control young cells (p=0.0001). Pft-treated old cells showed telomeres significantly longer than untreated ones (p= 0.0147). Control senescent fibroblasts showed telomeres significantly shorter than control young (p<0.0001) and old (p=0.0078) cells. Senescent cells chronically treated with pft and pft+siATRX showed telomeres significantly longer than control ones (p=0.0014 and p=0.0097).

## Discussion

During the present study, we observed several phenomena linked to the aging process of human fibroblasts. Obviously, we noticed telomere shortening (**Figure 7**), but also an increase of telomere damage (**Figure 4**). A drastic increase of TIF in senescent cells has already been observed (38). Moreover, we observed an increase of telomere cohesion, in agreement with (39) and in accordance with the fact that tankyrase (which is a poly-ADP-ribose polymerase that is required for resolution of telomere cohesion (40) decreases in senescent human fibroblasts (41).

Being widely used as a marker of ALT, we also investigated presence of APB. PML bodies are protein complexes involved in p53-associated apoptosis, DNA damage response, DNA repair and, in particular, HR (42). They show telomeric localization (i.e., APB) in ALT cells and are thought to host the HR machine responsible for recombination at telomeres (43). Our results are in agreement with those of (44), as they observed that APB are present in normal human fibroblasts and that their frequencies drastically increase in senescent cells. The circa two-fold increase of APB that we observed was paralleled by a two-fold increase of PML (**Figure 3**).

Interestingly, our data also show a decrease of HR (**Figure 4**) in aged cells, in agreement with Mao et al. (33). As a consequence of this, despite senescent cells had high levels of telomeric damage, this was not accompanied by an increase of telomeric recombination (**Figure 5**).

Silencing of ATRX in young cells significantly reduced RAD51 (**Figure 4**). Nonetheless, it also caused genomic and telomeric damage, which led to an increase of HR (RAD51+H2AX) and telomeric HR (T-SCE, **Figure 5**). In senescent cells, in which RAD51 was already drastically diminished by aging, silencing of ATRX caused a further reduction of RAD51 and an almost complete abrogation of telomeric recombination. Unexpectedly to us, siATRX treatment significantly decreased both PML and APB in young cells (and to a lesser degree in senescent cells). However, it was previously found that ATRX regulates PML expression and protects PML protein from proteasome-dependent degradation, and that inactivation of ATRX diminishes PML-NBs (45). It could be speculated that PML was less depressed in senescent cells compared to younger ones because in senescent fibroblasts the proteasome is less efficient (46). Moreover, chronic treatment with siATRX significantly decreased SAHF (**Figure 2**) and this may be the cause for the observed slight increase of proliferation, i.e. a slight delay of the onset of senescence.

Inhibition of p53 caused an increase of RAD51 both in young and senescent cells (**Figure 4**). This is in agreement with earlier studies showing that p53 binds to the promoter of *RAD51*, leading to the downregulation of RAD51 messenger RNA and protein (22). In young cells, in which telomeric damage is absent, p53 inhibition did not change T-SCE frequencies. On the contrary, in old and senescent cells (presenting high levels of telomeric damage), p53 inhibition drastically increased T-SCE frequencies (**Figure 5**). This confirms earlier works showing that p53 inhibition increases HR (21) and in our case, we showed that it increases telomeric HR. The same was observed in the first part of our work (35), in which we observed that p53 inhibition increased T-SCE frequencies in X-ray-treated young fibroblasts (which also have telomeric damage). Chronic p53 inhibition also produced a small but significant increase of telomere length (**Figure 7**) or, more correctly, a decrease of telomere shortening; it could be hypothesized that this may be ascribable to the increased telomeric recombination, thus reminiscent of ALT activity.

Silencing of ATRX combined with p53 inhibition in senescent cells (both short-time and chronically treated) determined an increase of APB and of the APB/PML ratio. Chronic treatment caused also a decrease of SAHF and a delay of senescence. Senescent cells treated with siATRX and pft (both for 3 days and chronically for 20 days) exhibited an increase of RAD51 and RAD51/H2AX foci (**Figure 4**). Similarly to senescent cells treated with pft alone, this increase of HR was mirrored also by an increase of T-SCE and, in the case of chronically treated cells, it also caused a decrease of telomere shortening.

As far as we know, no study on the effect of p53 inhibition and ALT activity has been conducted so far. On the other hand, the studies on the effects of ATRX silencing gave contradictory results: it did not activate an ALT phenotype in HCT-116 (47) and 22Rv1 cells (48), while it activated it in LAPC-4 cells (48). Similarly, telomerase inactivation led to ALT activation in surviving clones of H1299, SW39 (49), T24 (50), Hep-2 cells (51), but not in HT1080 (49). However, an examination of the p53 status of these cell lines shows that the ALT phenotype was obtained in p53-defective lines, while it failed in p53-proficient cells (**Table 1**). Interestingly, also infection of endothelial cells with Kaposi’s sarcoma virus, which encodes the p53-inhibiting protein LANA (52), led to an ALT phenotype (53). It should be also added that in the works in which spontaneously immortalized ALT-fibroblasts were obtained after ATRX-inactivation (47, 54) the cells were SV40-infected, thus p53-deficient. Moreover, upon depletion of ATRX in HeLa and HEK293 cells (which both have inactive p53), Scott et al. (55) found increased levels of telomeric H2AX, PML, POLD3 and decreased levels of telomeric BRCA2, a picture similar to the one seen in ALT models. The authors also found evidence of ATRX-mediated recruitment of SLF2 (a member of the SMC5/6 complex) to telomeres and found that the loss of these proteins causes an increase of T-SCE and “telomere exchanges” (a kind of T-SCE showing an exchange on a chromatid and a single signal on the other chromatid, see **Figure 5**). Thus, it seems that loss of ATRX induces an ALT phenotype only if it is also accompanied by loss of p53. It could be argued that this is due merely to the fact that ATRX loss is lethal and p53 loss allows the survival of ATRX-deficient cells. However, we have demonstrated that p53 inhibition increases recombination of damaged/dysfunctional telomeres and it was previously demonstrated that p53 inhibits HR independently from its cell-cycle blocking activity (24–26).

**Table 1:** ALT activation in different cell lines.

Cell line	Model	Telomerase activity	P53 status	ATRX status	ALT activation	T-SCE	C-circles	APB	Telomere heterogeneity	P53	ALT	Reference
H1299		yes	defective (mutated)	wild-type					no			43
	TERC KO	no	defective (mutated)		yes (survivors)		yes		yes			
SW39		yes	defective (mutated)	wild-type			no	no	no			43
	TERC KO	no	defective (mutated)	wild-type	yes (survivors)		yes	yes	yes			
HT1080		yes	wild-type	wild-type								43
	TERC KO	no	wild-type	wild-type	no survivors							
LAPC-4		yes	defective (mutated)	wild-type								42
	ATRX KO	yes	defective (mutated)	KO			yes	yes	yes			
	ATRX/TERC KO	no	defective (mutated)	KO	yes (survivors)		yes	yes	yes			
22Rv1		yes	ambiguous	wild-type			no	no	no			42
	ATRX KO	yes	ambiguous	KO	no		no	no	no			
HCT-116		yes	wild-type	wild-type				no				41
	ATRX KO	yes	wild-type	KO	no			no				
T-24		yes	defective (mutated)	wild-type								44
	TERT KO	no	defective (mutated)	wild-type	yes (survivors)			yes				
Hep-2		yes	defective (HPV-16)	wild-type								45
	TERT KO	no	defective (HPV-16)	wild-type	yes (survivors)	yes		yes				
EA.hy926		yes	wild-type	wild-type		no	no	no				47
	KHSV	reduced	defective (KHSV)	defective (KHSV)	yes	yes	yes	yes				
SLK		yes	wild-type	wild-type		no	no	no				47
	KHSV	reduced	defective (KHSV)	defective (KHSV)	yes	yes	yes	yes				

KHSV, Kaposi**’**s sarcoma virus. P53: green = proficient; red = deficient; brown = ambiguous. ALT: green = activated; red = not activated.

We showed that senescence leads to a phenotype that seems permissive for ALT activity, i.e. high levels of APB, telomeric damage and telomeric cohesion. However, being HR highly repressed, telomeric recombination, upon which the ALT machinery relies, is almost absent. Silencing of ATRX greatly increases telomeric recombination in young cells, but is not able to overcome senescence-induced HR repression. Conversely, inhibition of both p53 and ATRX leads to a phenotype reminiscent of some aspects of ALT activity, with a further increase of APB, a decrease of telomere shortening (and increased proliferation) and, above all, an increase of telomeric recombination. We think that further studies on the establishment of ALT in primary cells facing senescence (rather than silencing genes in telomerase-positive cancer cells) will be useful for elucidating the activation of this telomere maintenance mechanism, which is particularly important in the field of the study of sarcomas, since these show the highest prevalence of ALT (9).

## Data availability statement

The original contributions presented in the study are included in the article/**Supplementary Material**. Further inquiries can be directed to the corresponding author.

## Ethics statement

Ethical approval was not required for the studies on humans in accordance with the local legislation and institutional requirements because only commercially available established cell lines were used.

## Author contributions

IU: Conceptualization, Formal Analysis, Investigation, Methodology, Writing – original draft, Writing – review & editing. JM: Investigation, Writing – original draft. AS: Conceptualization, Supervision, Writing – review & editing.
