# Supplementary material for: Effects of p53 and ATRX inhibition on telomeric recombination in aging fibroblasts
Source: Front Oncol. 2024 Jan 25;14:1322438. doi: 10.3389/fonc.2024.1322438 (PMC10850245; doi:10.3389/fonc.2024.1322438)
Supplement: Supplementary file 1 [file DataSheet_1.docx]

**Effects of p53 and ATRX inhibition on telomeric recombination. Part II: Impact on aging primary fibroblasts**

***Supplementary material***

**P53 inhibition**

P53 inhibition was evaluated by measurement of p21 induction (using immunofluorescence staining) after X-irradiation. Briefly, cells were fixed with ice-cold methanol 4 and 24 hours after irradiation, then blocked in BSA for 30 minutes and then incubated for one hour at 37°C with mouse anti-p21 antibody (Santa Cruz Biotechnology, USA). Cells were then incubated with secondary anti-mouse Alexa 488 (Invitrogen) for 1 hour at 37°C. Coverslips were mounted with DAPI in antifade solution. Cells were analyzed with an Axio Imager M1 fluorescent microscopy (Carl Zeiss, Jena, Germany). Percentage of p21-positive cells was evaluated analyzing 300 cells per replicate. Three independent experiments were performed.


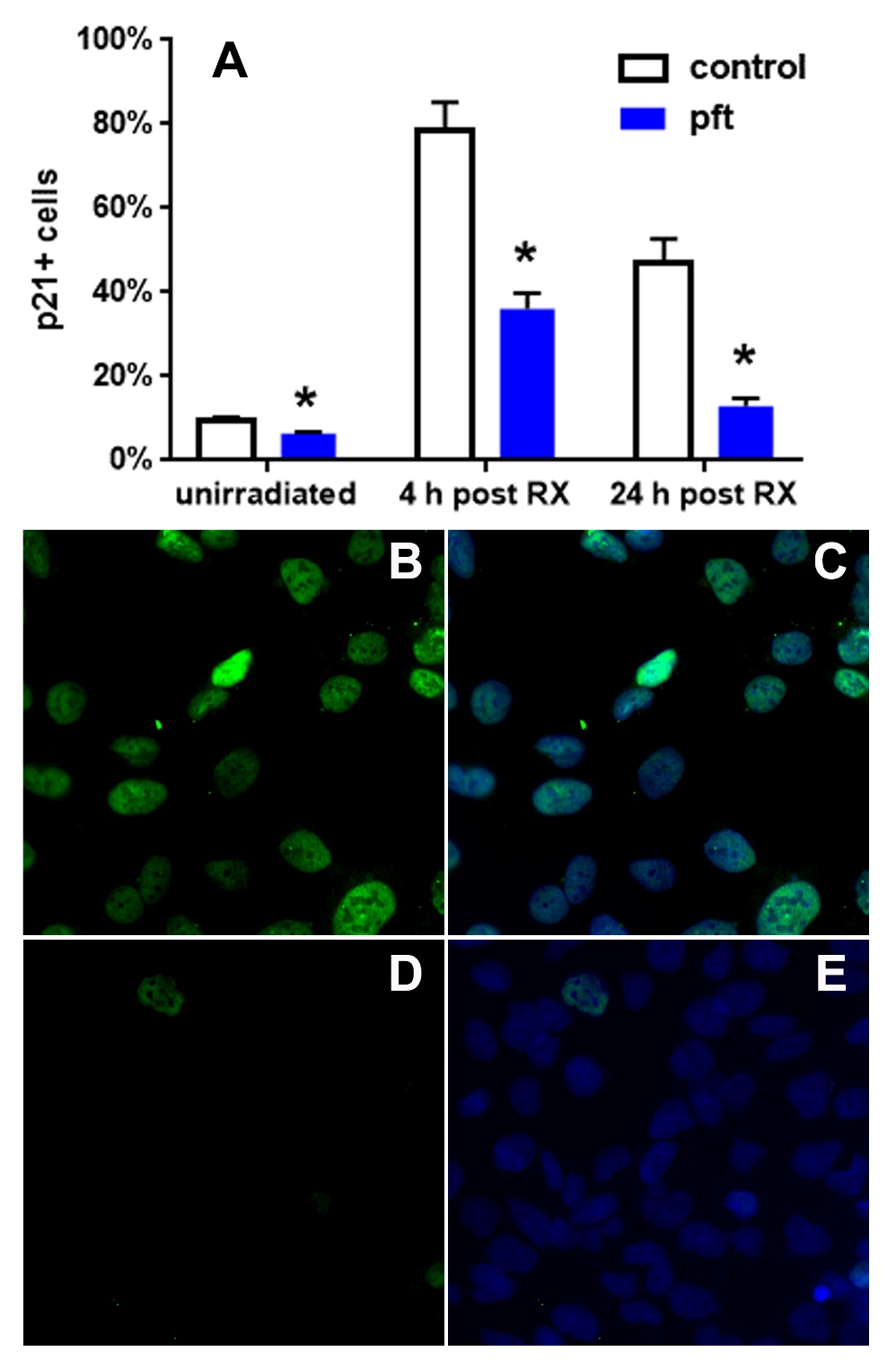


**Figure S1 – Effectiveness of P53 inhibition.** A: Frequencies of p21-positive cells in untreated and pft-treated samples. The results are expressed as means ± S.E.M. (n = 3) and were evaluated by two sample t test. The level of significance was established at p<0.05. *: significant compared to paired control sample. B-C: X-irradiated cells. D-E: Pft-treated X-irradiated cells. B, D: Images acquired with Alexa 488 filter. C, E: Merge of Alexa 488 and DAPI filters. Cells are stained with Alexa 488-conjugated anti-p21 antibody and DAPI.

**ATRX silencing**

SiRNA transfection is described in the Material and methods of the main text. In order to test the efficacy of ATRX silencing, we performed Western blotting on whole cell extracts. Cells were lysed in RIPA Buffer, and protease inhibitors. Protein extracts (50 μg) were loaded on an SDS-PAGE and transferred onto a polyvinylidene fluoride (PVDF) membrane (pore size 0.45 μm; Immobilion-P, Millipore). Filters were blocked with 3% BSA dissolved in Tris Buffered Saline (TBS) with 0.05% Tween-20 (TBS-T) for 1 hour at RT. Membranes were then incubated at 4 °C overnight with the following primary antibodies: Vinculin (#v9131, Sigma-Aldrich), ATRX (#HPA001906, Sigma-Aldrich). Finally, membranes were incubated 1 hour at room temperature with the appropriate HRP-conjugated secondary antibody (Bio-Rad Laboratories, USA). Proteins were visualized using ClarityTM Western ECL substrates (Bio-Rad Laboratories). Images were acquired using the ChemiDoc™ Imaging system (Bio-Rad).

SiRNA treatment drastically reduced ATRX protein level (**Figure S2A**). Further proof of ATRX silencing was obtained by immunofluorescence with anti-ATRX antibody (Santa Cruz Biotechnology, USA), which showed total lack of ATRX foci in siATRX-treated samples (**Figure S2E**).


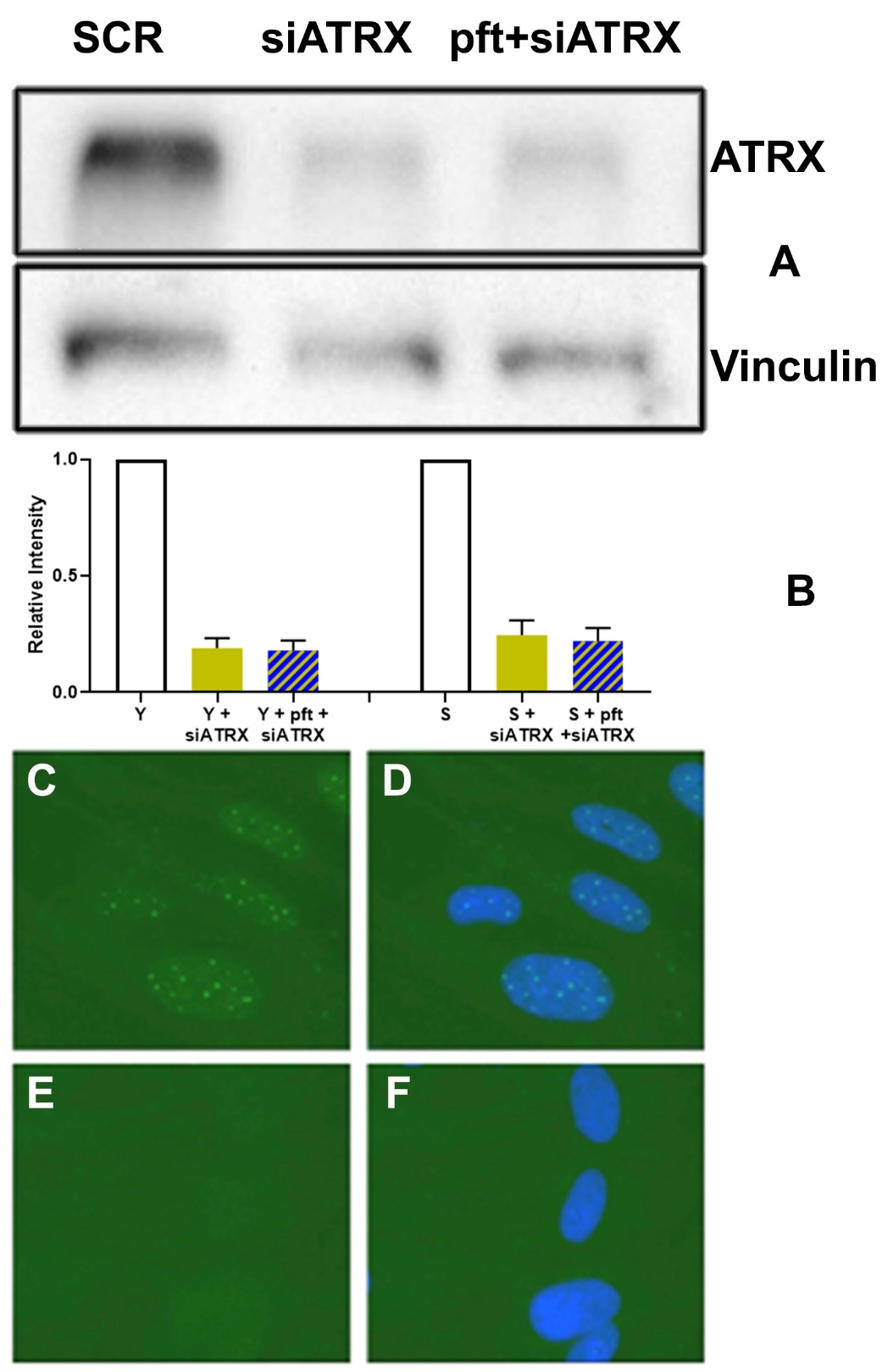


**Figure S2 – Effectiveness of ATRX silencing.** A: Western immunoblotting showing efficiency of ATRX silencing. B: Bar diagram representing Western blot analysis of ATRX protein levels (relative to vinculin); Y: young; S: senescent. C-F: Immunofluorescent staining of ATRX foci. C-D: Scramble-treated cells. E-F: SiATRX-treated cells. C, E: Images acquired with Alexa 488 filter. D, F: Merge of Alexa 488 and DAPI filters. Cells are stained with Alexa 488-conjugated anti-ATRX antibody and DAPI.

**Aging-related markers**

**A B**


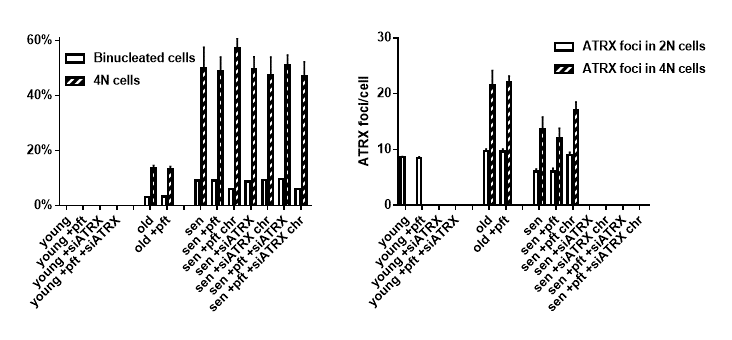


**Figure S3.** A: Percentages of binucleated and polyploid (4N) cells. B: Frequencies of ATRX foci in diploid (2N) and polyploid (4N) cells. Y: young; O: old; S: senescent; ctrl: controls; + pft chron.: chronically treated with pft; + siATRX chron.: chronically treated with siATRX; + pft + siATRX chron.: chronically treated with pft and siATRX.

**Single-channel images**


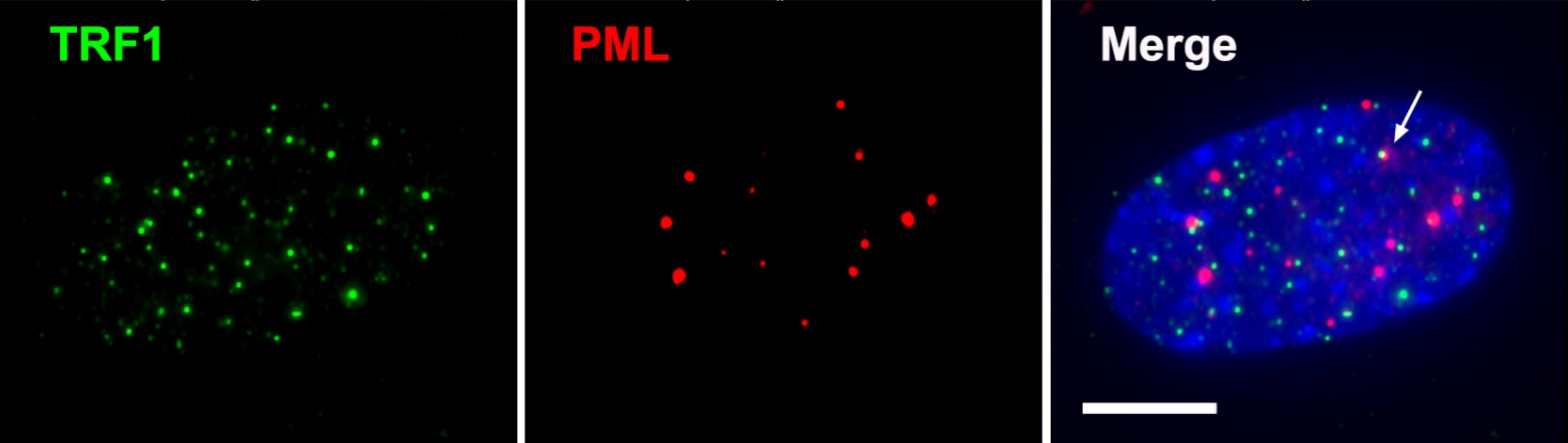


**Figure S4 – APB immunofluorescence.** Single-channel photos of Figure 3. Arrow shows colocalization. Scale bar, 5 µm.


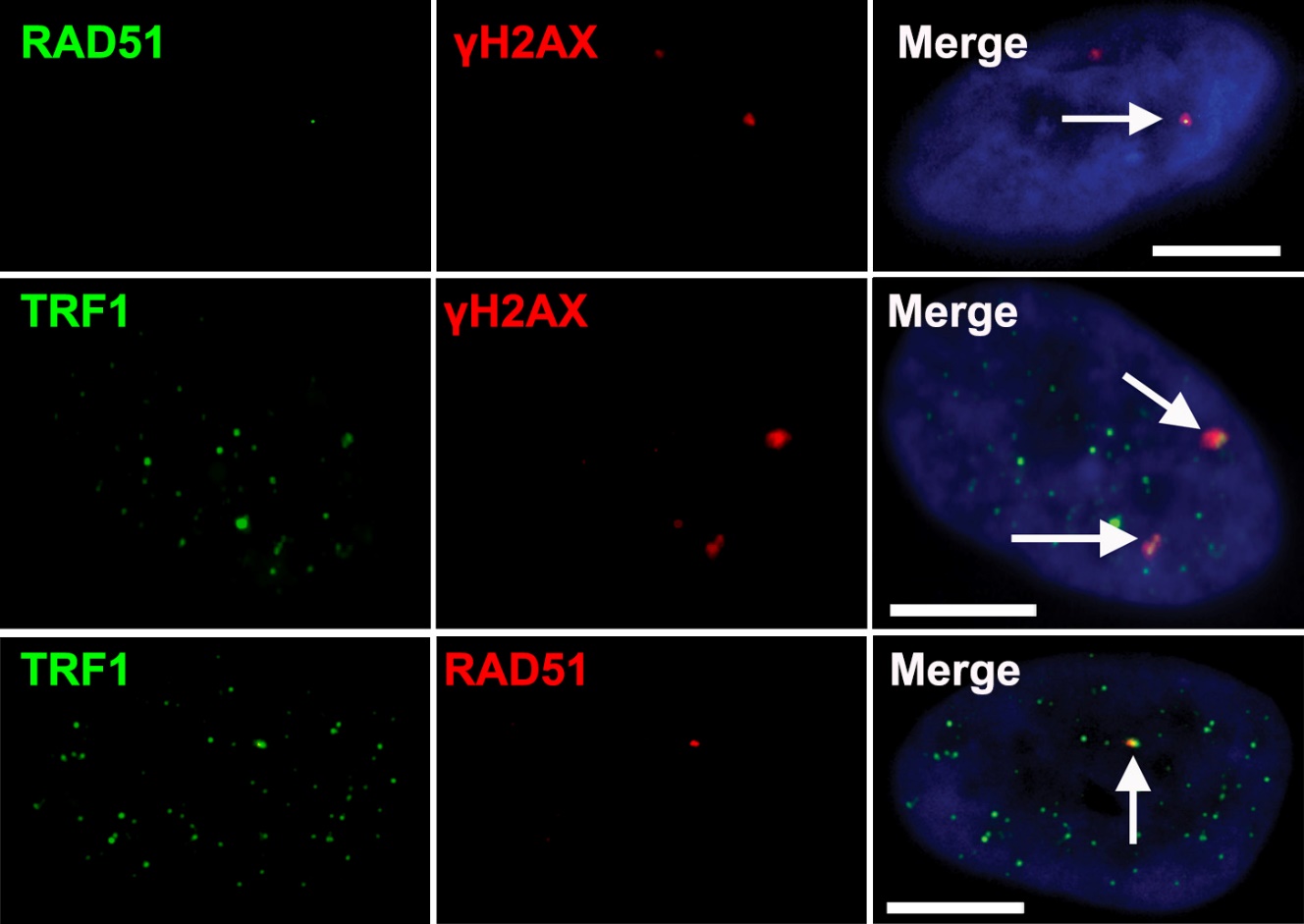


**Figure S5 – DNA damage and repair foci.** Single-channel photos of Figure 4. Arrows show colocalizations. Scale bar, 5 µm.


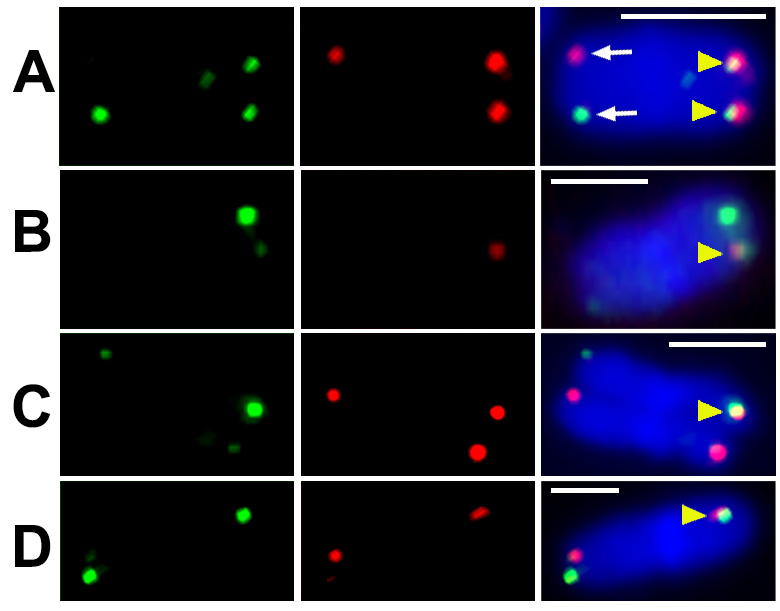


**Figure S6 – Telomeric sister chromatid exchanges (T-SCE).** A-D: Single-channel photos of Figure 5C-F. Arrows show normal telomeres, yellow arrowheads show double signals (T-SCE). Scale bar, 2 µm.


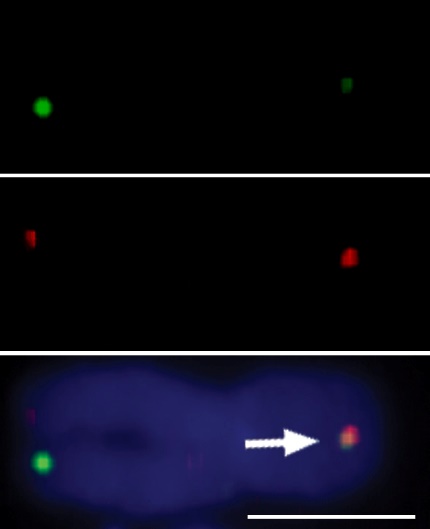


**Figure S7 – Sister telomere fusion.** Single-channel photos of Figure 6. Scale bar, 2 µm.

**Nucleoplasmic bridges and dicentrics**

To further confirm observations on sister telomere fusions (STF), we analysed nucleoplasmic bridges (NPB) in binucleated cells discriminating between telomere-containing NPB (NPB-TRF1+, which derive from STF and telomere-containing dicentrics) and telomere-free NPB (NPB-TRF1-, which derive only from telomere-free dicentrics). Binucleated cells were obtained by adding cytochalasin B in young cells; in senescent cells binucleated cells were spontaneously formed (**Figure S8A**); in old fibroblasts, spontaneous binucleated cells were too few and the cell cycle time too long for cytochalasin B treatment, therefore this test was not performed. Similarly to STF, young cells treated with siATRX and pft+siATRX showed an increase of NPB-TRF1+ (**Figure S8A**), being statistical significant in pft+siATRX-treated cells (p=0.0398). In senescent cells there was a significant increase of NPB-TRF1+ (p=0.049) compared to the young ones. Although all treatments of senescent cells induced increases of NPB-TRF1+, none of them was significant. No NPB-TRF1- was present in young cells, whereas there was a significant increase in senescent cells (p=0.0016); also in this case, increases of NPB-TRF1- due to treatments were not significant.

No young fibroblasts showed either telomere-containing (Dic-tel+) or telomere-free dicentrics (Dic-tel-, **Figure S8B**). Among senescent fibroblasts, only short- and chronically-pft-treated and chronically-pft+siATRX-treated cells showed Dic-tel+, representing a significant increase (p=0.0045, p=0.0045 and p=0.0257, respectively) compared to senescent untreated cells. Concerning Dic-tel-, senescent fibroblasts showed a significant increase compared to young cells (p=0.0226). No treatment in senescent cells significantly changed Dic-tel-.


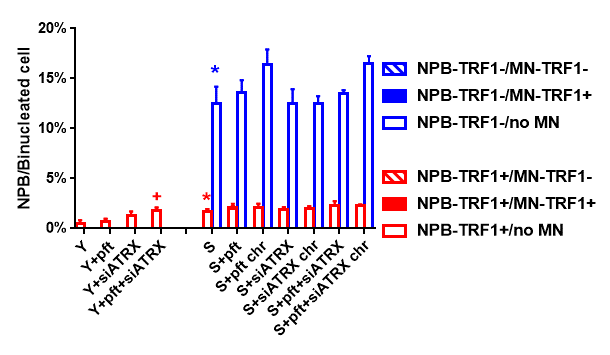

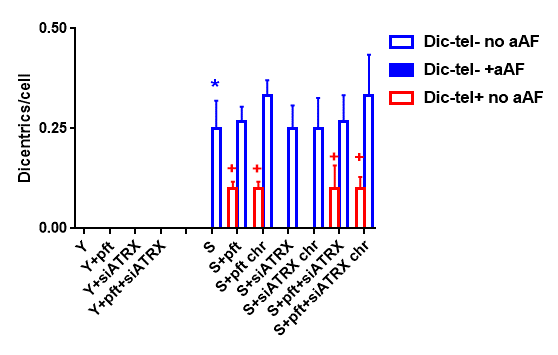


**B**

**A**

**Figure S8.** A: Percentages of Nucleoplasmic bridges (NPB) in binucleated cells. NPB are classified as with (TRF1+) or without (TRF1-) telomere and accompanied by a telomere-containing (MN-TRF1+) or telomere-free (MN-TRF1-) micronucleus or without micronucleus (no MN). B: Percentages of Dicentrics in chromosome spreads. Dic-tel+ no aAF: telomere-containing dicentrics without accompanying acentric fragment; Dic-tel- no aAF: telomere-free dicentrics without accompanying acentric fragment; Dic-tel- +aAF: telomere-free dicentrics with accompanying acentric fragment. *: significant compared to young cells; +: significant compared to untreated cells.
